# 454 Transcriptome Sequencing Suggests a Role for Two-Component Signalling in Cellularization and Differentiation of Barley Endosperm Transfer Cells

**DOI:** 10.1371/journal.pone.0041867

**Published:** 2012-07-25

**Authors:** Johannes Thiel, Julien Hollmann, Twan Rutten, Hans Weber, Uwe Scholz, Winfriede Weschke

**Affiliations:** 1 Leibniz-Institut für Pflanzengenetik und Kulturpflanzenforschung (IPK), Gatersleben, Germany; 2 Botanisches Institut, Christian-Albrechts-Universität, Kiel, Germany; Centro de Investigación y de Estudios Avanzados del IPN, Mexico

## Abstract

**Background:**

Cell specification and differentiation in the endosperm of cereals starts at the maternal-filial boundary and generates the endosperm transfer cells (ETCs). Besides the importance in assimilate transfer, ETCs are proposed to play an essential role in the regulation of endosperm differentiation by affecting development of proximate endosperm tissues. We attempted to identify signalling elements involved in early endosperm differentiation by using a combination of laser-assisted microdissection and 454 transcriptome sequencing.

**Principal Findings:**

454 sequencing of the differentiating ETC region from the syncytial state until functionality in transfer processes captured a high proportion of novel transcripts which are not available in existing barley EST databases. Intriguingly, the ETC-transcriptome showed a high abundance of elements of the two-component signalling (TCS) system suggesting an outstanding role in ETC differentiation. All components and subfamilies of the TCS, including distinct kinds of membrane-bound receptors, have been identified to be expressed in ETCs. The TCS system represents an ancient signal transduction system firstly discovered in bacteria and has previously been shown to be co-opted by eukaryotes, like fungi and plants, whereas in animals and humans this signalling route does not exist. Transcript profiling of TCS elements by qRT-PCR suggested pivotal roles for specific phosphorelays activated in a coordinated time flow during ETC cellularization and differentiation. ETC-specificity of transcriptionally activated TCS phosphorelays was assessed for early differentiation and cellularization contrasting to an extension of expression to other grain tissues at the beginning of ETC maturation. Features of candidate genes of distinct phosphorelays and transcriptional activation of genes putatively implicated in hormone signalling pathways hint at a crosstalk of hormonal influences, putatively ABA and ethylene, and TCS signalling.

**Significance:**

Our findings suggest an integral function for the TCS in ETC differentiation possibly coupled to sequent hormonal regulation by ABA and ethylene.

## Introduction

Crop seeds develop in distinct successive phases of tissue-specific differentiation [1]. After double fertilization, endosperm development of barley starts with divisions of nuclei without cell wall formation resulting in the formation of the endosperm coenocyte. Cell fate specification occurs already in the endosperm coenocyte [2] and is proposed to be determined by surface position rather than signalling from maternal tissues [3]. Around 3–4 days after flowering (DAF), cellularization starts and is finished within 24–36 hours. Endosperm cellularization is accompanied by the differentiation of the nucellar projection (NP), that part of the nucellus facing the main vascular bundle. Differentiation of the endosperm starts in the outermost cell row adjacent to NP generating the highly specific endosperm transfer cells (ETC) and will be completed after differentiation of the aleurone cells. During this time (5 to 10 DAF) transcriptional reprogramming indicates transition of the caryopsis into a storage product accumulating organ.

Developing seeds are sink tissues depending on nutrient supply from vegetative tissues. Incoming assimilates, delivered by the vascular bundles, are released from the maternal grain part by NP, which is responsible for transfer but also for interconversion of assimilates, especially amino acids [4]. Released assimilates are taken up by ETCs and supplied to the endosperm. Differentiation and function of ETCs and NP have to be coordinated with changing endosperm sink strength and undergo hormonal regulation [5]. Beside the importance for assimilate delivery ETCs are responsible for the regular differentiation of the endosperm resulting in the two major tissues, starchy endosperm and aleurone. Evidence for the pivotal role of ETCs in differentiation events of the endosperm came from studies with the barley endosperm mutant *seg8*. *Seg8* is defective in cellularization and differentiation of ETCs and revealed a strongly reduced or completely missing middle part of the endosperm resulting in a reduction of seed weight up to 70% [6]. Defects in cellularization of ETCs and subsequently, endosperm differentiation have been attributed to altered ABA levels and ABA effects [7].

Sequence information for barley is largely generated by EST sequencing projects since the barley genome has not been sequenced completely until now. HarvEST:barley (http://harvest.ucr.edu/) assembly 35 represents the most comprehensive EST collection of barley containing 444,652 sequences from different varieties, organs/tissues and treatments. ESTs from barley grain tissues were generated from non-normalized cDNA libraries representing the maternal and filial grain part after rough manual dissection. Thus, the EST collection mainly contains highly abundant mRNA species and is less specific for small tissue regions embedded in barley grains. The recent development of next-generation sequencing (NGS) technologies provides new opportunities to acquire sequence information independent of genomic resources. As it has been shown for isolated cells of the maize shoot apical meristem, the combination of 454 transcriptome sequencing with laser-assisted microdissection has the potential to capture rare and highly specialized transcripts which are underrepresented in existing EST collections [8].

Here, we report the 454 transcriptome sequencing of cDNA generated from ETC regions at distinct developmental stages, just before the beginning of cellularization of the endosperm until the transition phase of grain development, when differentiation of the endosperm tissues is nearly completed. The 454 sequencing approach gained a surprisingly high amount of new sequence information. About 40% of the generated contigs were not found in public databases of barley; among them, about 8,000 contigs could be functionally annotated or attributed to assigned functions. The high number, mRNA abundance and localization of elements of the two-component signalling (TCS) system in barley ETCs indicate an outstanding role of these signalling pathways for regular specification and differentiation of ETCs and subsequently, for endosperm development.

## Results

### Generation of tissue-specific cDNA libraries and 454 sequencing

**Figure 1:**
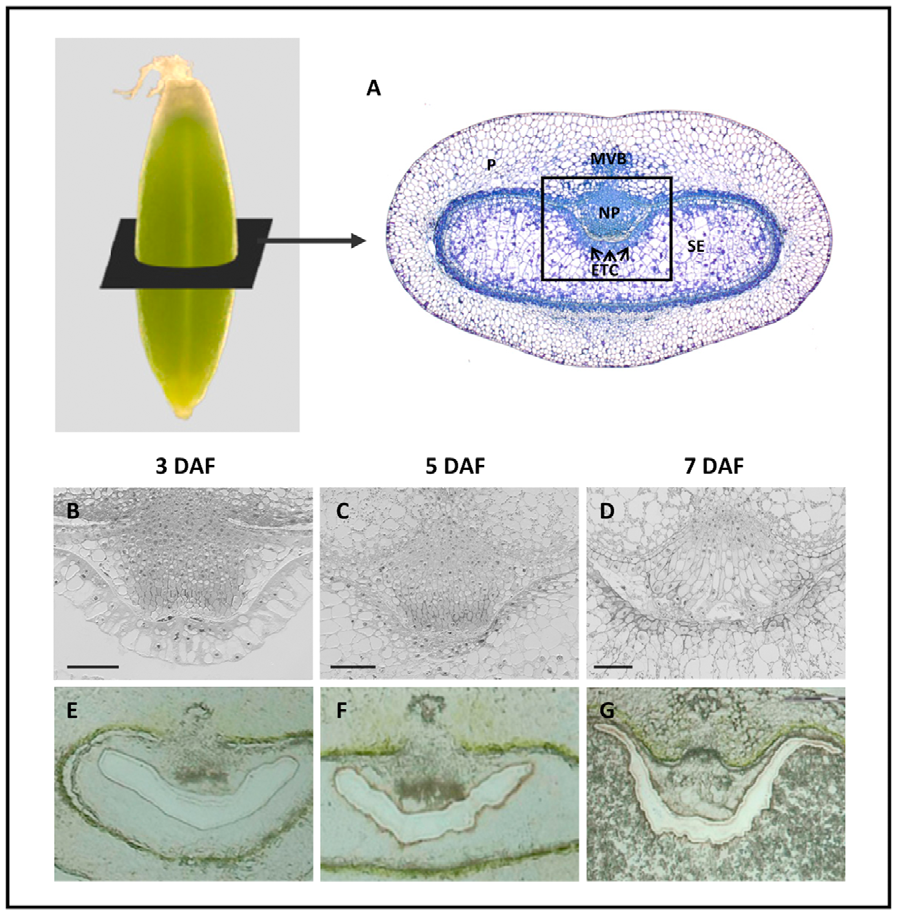
Representation of barley grain tissues, morphology of differentiating ETCs and cryosections after microdissection. (A) Median cross-section of a barley grain at 7 DAF depicting the main grain tissues. (B–D) ETC region in detail at 3, 5 and 7 DAF. Note that in (B) details of a barley grain between 3 and 4 DAF are represented to illustrate the beginning of the cellularization process. (E–G) Representative examples of cryosections after isolation of the ETC region. ETC: endosperm transfer cells, MVB: main vascular bundles, NP: nucellar projection, P: pericarp, SE: starchy endosperm. Bars represent 100 µm.

Tissues of barley grains (Fig. 1A) and details of ETC morphology during differentiation are presented in Fig. 1. At 3/4 DAF, the syncytium in opposite to the NP, which will later differentiate into ETCs, starts to cellularize (Fig. 1B). At 5 DAF, ETCs have been cellularized and simultaneously, the endosperm vacuole is filled with cells (Fig. 1C). During further differentiation characteristic cell wall thickenings became apparent in ETCs at 7 DAF (Fig. 1D). ETCs at 3, 5 and 7 DAF have been cut out and isolated from barley grain cross-sections via laser microdissection and pressure catapulting (LMPC). For tissue preparation we used cryosections without any chemical pre-treatment to preserve RNA quality while providing morphology for precise targeting of cells (Fig. 1. E–G).

After RNA extraction, mRNA amplification and quality assessment of antisense RNA (Figure S1) populations of each developmental stage were pooled. GS FLX Titanium sequencing has been conducted by GATC Biotech (Konstanz, Germany). From the pooled cDNA library 1.23 million 454 reads with an average read length of 269 nucleotides (nts) and an average trimmed read length of 248 nts were produced.

### Assembly of 454 sequences

After processing of the 454 reads (details in the method section) the CAP3 assembler produced 42,086 contigs with a mean contig size of 505 bp and a N50 value of 531. To confirm the reliability of the procedure the results were crosschecked with a MIRA-based assembly strategy yielding similar results (data not shown). To ensure that the contigs represent barley sequences and to exclude technical errors or contaminations, CAP3 contigs were mapped against a whole genome shotgun (WGS) assembly of the barley variety ‘Morex’ (30× coverage). By BlastN analysis (<1E-3) 87.6% of the sequences were found in the genomic data set (http://pgrc.ipk-gatersleben.de/blast/barlex/) confirming 454 contigs as barley sequences.

### Annotation of 454 contigs by comparison to protein and plant databases

To predict the function of the 42,086 contigs generated from the ETC transcriptome, contigs were compared to different databases by BlastX search (<1E-10, Table S1). At most 50% of the sequences got a hit in the UniProtKB/TrEMBL and the least specific NCBI nr database whereas only one fourth of the contigs got a hit in the accurately curated protein database UniProtKB/Swiss-Prot (Table 1). This is inasmuch remarkable, because the global nr database contains entries from a wide variety of databases of all organisms, which are publicly available. Similar results were obtained by comparisons to plant-specific databases: around 50% of the contigs were found in the Rice All-cDNA collection and in the v1.2 release of coding sequences (CDS) of the genome database of *Brachypodium distachyon*. In the TAIR9 release of the Arabidopsis cDNA collection only 15,988 (38%) of the contigs got a hit.

**Table 1:** Comparison of ETC transcriptome data to general and plant-specific databases.

No. contigs	database	hits BlastX (<1E-10)	hits (%)
42,086	UniProtKB/Swiss-Prot	11,477	27.3
“	UniProtKB/TrEMBL	21,085	50.1
“	NCBI nr_pep	21,416	50.9
“	TAIR9_cDNA/Arabidopsis	15,988	38.0
“	MIPS_CDS v1.2/Brachypodium	19,768	47.0
“	Rice Genome Annotation_All_cDNA	20,428	48.5

### Mapping of 454 sequences to a global collection of barley ESTs

**Figure 2:**
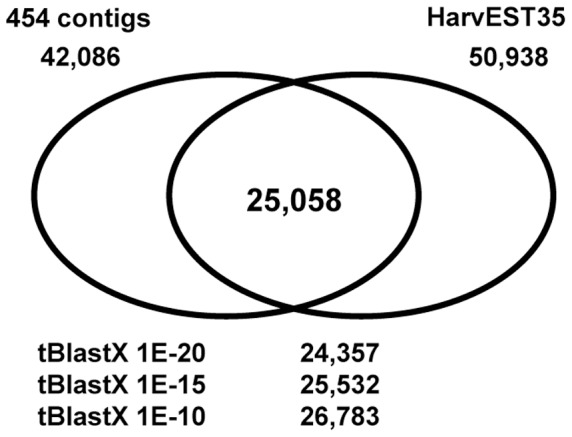
Comparison of 454 sequences with public barley sequence information. Venn diagram displays the number of overlapping sequences between ETC-454 contigs and sequences of the HarvEST assembly 35 (BlastN <1E-20). Commonalities using tBlastX searches with different stringencies are given at the bottom.

HarvEST assembly 35 represents the most comprehensive sequence collection for barley composed of 444,652 ESTs which have been assembled into 28,000 contigs and 22,938 singletons. The ETC-454 contigs were compared to the 50,938 assembled sequences of HarvEST35. As shown in Figure 2 (Table S2), sequence comparison by BlastN using an e-value cut-off of 1E-20 resulted in 25,058 overlapping sequences, which in turn revealed that 17,028 of the 454 sequences (∼40%) are not present in HarvEST35. Less stringent search algorithms do not have an effect on the recovery of ‘unknown’ sequences (tBlastX 1E-20 to 1E-10: 42%-36%). From these unexpected results it can be concluded that the LMPC-based 454 sequencing uncovered a high number of up-to-now unknown transcripts. This correlates to the specificity of this small tissue region and to the strong under-representation of ETC-expressed sequences in barley EST collections.

**Figure 3:**
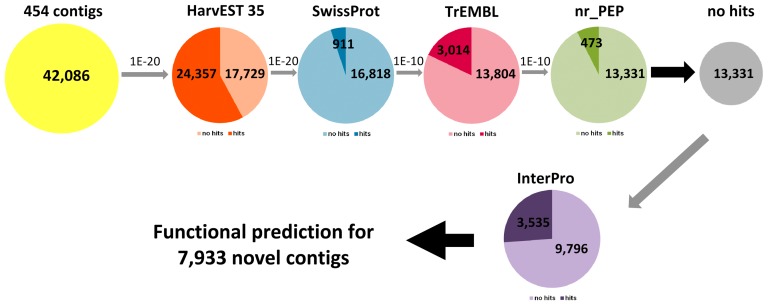
The multilevel process of filtering for functional assignment of ETC transcripts not present in HarvEST35. Dark shaded regions in pie charts indicate the number of hits in databases whereas light coloured regions display the number of transcripts not fulfilling the respective similarity criteria. Non-hitting sequences were further compared to the next protein database with decreasing stringencies (BlastX cut-offs 1E-20 to 1E-10). InterPro search for functional domains was employed for the sequences without sufficient similarity.

To characterize these novel transcripts more precisely, we performed a multilevel process of filtering from high to low stringency concerning similarity criteria and the specificity of the different databases (Fig. 3, Table S3). Starting from 42,086 contigs, tBlastX searches were performed against HarvEST35 with an e-value cut-off <1E-20, the non-hitting sequences were further compared to the next databases with increasing cut-offs (1E-20 to 1E-10). The dark shaded regions in pie charts illustrate the hits in the database whereas the light coloured regions indicate the number of sequences owing unsufficient similarity, which were afterwards compared to the next database. From the 17,729 contigs which are not present in HarvEST35, 4,398 contigs could be functionally annotated by comparison to the different protein databases converging to more lenient conditions (nr database, 1E-10) whereas 13,331 contigs remained in the ‘no hit’ category (Fig. 3). These contigs were further analyzed by search for functional domains using the InterPro database. For 3,535 contigs at least one functional domain has been identified in one of the six predicted open reading frames (ORFs). Collectively, 7,933 contigs, which are transcriptionally activated in differentiating ETCs and have not been available in public databases, were annotated or could be attributed to an assigned function. We will use the term ‘new ETC-specific sequences’ to refer to this part of the ETC transcriptome. In total, 32,290 contigs of the ETC transcriptome were functionally assigned whereas 9,796 contigs gave no information with regard to predicted functions.

### A multitude of transcripts involved in two-component signalling (TCS) was detected in barley ETCs

Signal transduction via the two-component signalling (TCS) system is deemed to play a role in early differentiation events of endosperm transfer cells in cereals [9]–[10]. The TCS represents a multi-step phosphorelay involving phosphorylation of His and Asp residues of proteins in a modular arrangement. During evolution, a third component, which transfers the phosphate group from the receptor to the regulatory element, has been integrated in the TCS of plants and fungi. Multi-step phosphorelays in plants are composed of a (usually) membrane-bound histidine kinase (HK), a histidine-containing phosphotransfer protein (HPt) and a separate response regulator (RR). A variety of processes in plants is influenced through the phosphorelay, usually by hormonal regulation [11].

Using rice TCS sequences [12], BlastX search in amino acid sequences inferred from ETC-454 contigs revealed that 40 genes probably being part of the TCS are expressed in ETCs, covering all elements and subgroups of this signalling pathway (listed in Table 2). Wherever applicable, sequence information of 454 contigs was complemented with EST resources from HarvEST35 and a recently published database of full-length cDNA libraries from spring barley (http://barleyflc.dna.affrc.go.jp/hvdb/index.html) as well as genomic information generated by BAC and Whole Genome Shotgun (WGS) sequencing of barley varieties at the IPK Gatersleben (http://pgrc.ipk-gatersleben.de/blast/barlex/, [13]). In general, transcripts are assigned according to sequence homology and functional domains and indicated gene functions should be considered as ‘putative’.

**Table 2:** A list of putative TCS components found in the transcriptome of differentiating barley endosperm transfer cells (ETCs) by 454 sequencing.

Signalling components	Gene Symbol	Contig ID	Domains$	Family	Length	Similarity to Rice (%)#
Histidine kinases	HvERS1	13113, 14069,	GAF, HK	Ethylene receptor	635 aa§	90
	HvERS2	17016, 19283	GAF, HK	Ethylene receptor	630 aa§	87
	HvERS3	29901	GAF, HK	Ethylene receptor	637 aa§	88
	HvETR1	c_321	HK (H-Q), REC	Ethylene receptor	796 aa§	79
	HvETR2	41784	GAF, HK, REC	Ethylene receptor	781 aa§	81
	HvERS/ETR	9443, 24802	GAF	Ethylene receptor	357 aa	85
	* ^,^ **HvHK1	1313	HK, REC	HK	1088 aa§	52
	* ^,^ **HvHK2	27729	HK, REC	HK	948 aa§	78
	HvHK3	6273	HK	HK	540 aa	50
	**HvHK4	27825	CHASE, HK, REC	Cytokinin receptor	986 aa§	84
	HvHK5	12351	CHASE, HK, REC	Cytokinin receptor	1019 aa§	77
HPt elements	HvHP1	c_2558	HPt	HPt	148 aa§	60
	**HvHP2	20816	HPt	HPt	143 aa§	60
	HvHP3	19623	HPt	HPt	141 aa§	82
	HvPHP1	8606	HPt (H-R)	Pseudo-HPt	130 aa	52
	HvPHP2	10701	HPt (H-Q)	Pseudo-HPt	151 aa§	91
	* ^,^ **HvPHP3	15857	HPt (H-Q)	Pseudo-HPt	127 aa	45
Response Regulators	* ^,^ **HvRR1	11896	REC	Type-A	143 aa	94
	HvRR2	11144	REC	Type-A	287 aa	81
	HvRR3	10973	REC	Type-A	173 aa§	75
	* ^,^ **HvRR4	c_601, c_1687	REC	Type-B	128 aa	73
	HvRR5	2421	REC, MYB	Type-B	684 aa§	84
	* ^,^ **HvRR6	4929	REC, MYB	Type-B	562 aa	41
	* ^,^ **HvRR7	4245, 28937	REC, MYB	Type-B	602 aa	41
	* ^,^ **HvRR8	c_1259	REC, MYB	Type- B	674 aa§	37
	* ^,^ **HvRR9	14612	REC, MYB	Type- B	653 aa	35
	* ^,^ **HvRR10	4962, 35573, 17813	REC, MYB	Type- B	553 aa	52
	HvRR11	c_1528, 3508	REC, MYB	Type- B	623 aa§	75
	* ^,^ **HvRR12	44000	REC	Type- B	126 aa	69
	* ^,^ **HvRR13	13056	REC, MYB	Type- B	621 aa	44
	* ^,^ **HvRR14	3205	REC, MYB	Type- B	610 aa	35
	HvRR15	c_473	REC	Type- C	134 aa§	56
	HvRR16	c_1495	REC	Type- C	134 aa§	58
	* ^,^ **HvRR17	13700	REC	Type- C	92 aa	63
	* ^,^ **HvRR18	6171	REC	Type- C	133 aa§	53
	**HvPRR1	29831, c_2383 c_1724	REC (D-V)	Pseudo-RR	182 aa	42
	**HvPRR2	c_1185, 15192	REC (D-A), MYB	Pseudo-RR	347 aa	42
	HvPRR3	15567	REC(D-E), CCT	Pseudo-RR	759 aa§	69
	HvPRR4	21515	REC(D-E), CCT	Pseudo-RR	604 aa§	66
	HvPRR5	30930	REC(D-E), CCT	Pseudo-RR	522 aa§	76

Sequence information was complemented using public available EST databases and genomic sequences generated at the IPK Gatersleben. Prediction of coding sequences and protein sequences was done using FGENESH (http://linux1.softberry.com) and Augustus gene prediction (http://augustus.gobics.de) web-based interfaces.

$ noted domains are histidine kinase domain (HK), receiver domain (REC), GAF domain which is implicated in ethylene binding, CHASE domain for cytokinin binding, conserved histidine-containing phosphotransfer domain (HPt), Myb-like DNA binding domain (MYB), CCT motif found in clock proteins (CCT); brackets mark diverged domains and indicate changed amino acids,

* not present in HarvEST 35,

** not present in full-length cDNA collection of spring barley,

# inferred amino acid sequences were compared with protein sequences computed in the Rice Genome Annotation project using the BlastP tool with the default settings,

§ full-length protein sequence.

#### Three types of histidine kinase transcripts are found in ETCs

Hybrid histidine kinases (HKs) can be divided into three subgroups: (1) the ethylene receptor family, containing additionally to the histidine kinase domain (HK) and receiver domain a so-called GAF domain, which is implicated in ethylene binding, (2) proteins of the cytokinin receptor family which include an additional CHASE domain that functions in cytokinin binding, and (3) the ‘classical’ histidine kinases, initially identified as osmosensors [14]–[15]. Eleven sequences encoding HKs have been uncovered in the ETC transcriptome, among them six putative ethylene receptors, two putative cytokinin receptors and three transcripts showing similarity to histidine kinases of rice and Arabidopsis (Table 2). Numbering of barley gene symbols was performed in relation to the respective TCS elements of rice and Arabidopsis.

**Figure 4:**
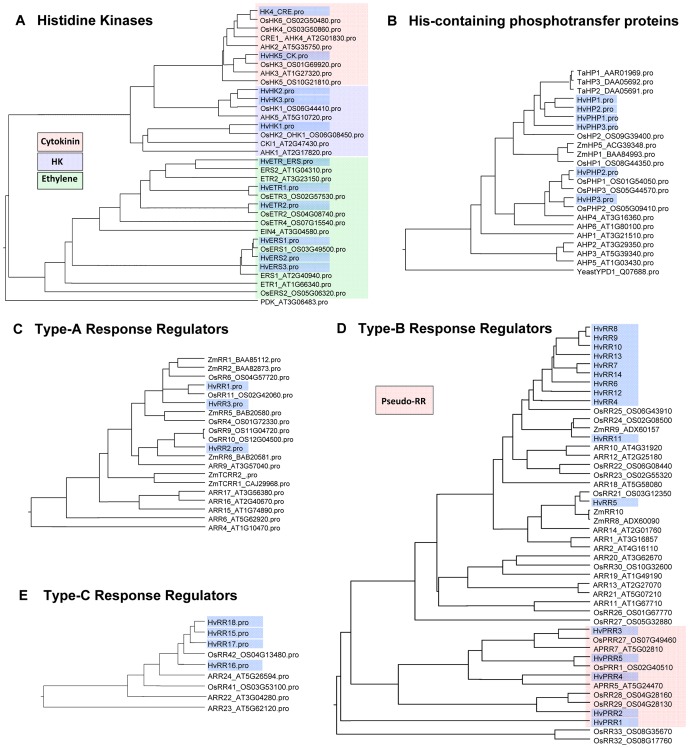
Phylogenetic relationship of barley TCS elements and counterparts of *Arabidopsis thaliana* and *Oryza sativa*. Phylogenetic trees are based on inferred amino acid sequences from complemented barley 454 contigs (see information in Table 2) and full-length amino acid sequences adopted from different databases (TAIR release 9, Rice Genome Annotation project and NCBI Protein database). Multiple alignments were performed using ClustalW algorithm with BLOSUM protein weight matrix (DNAstar software). Barley sequences are highlighted by blue boxes. Elements from other species are identified by chromosome loci or GenBank accession number. (A) Histidine kinases, colours indicate different subgroups. Arabidopsis PDK was used as outgroup. (B) HPt elements, protein sequences of *Zea mays* and *Triticum aestivum* were additionally included in the alignment. Yeast HPt protein YPD1 was used as outgroup. (C–E) Type-A, -B, -C response regulators, amino acid sequences of selected maize response regulators were included in the alignment. For reasons of simplicity not all Arabidopsis and rice elements were included in the phylogenetic trees.

Ethylene receptors can be divided into the ETR and the ERS subgroup, which is characterized by a missing receiver domain. Three protein sequences inferred from ETC-transcripts cluster together with ERS1 of rice and Arabidopsis (Fig. 4A). Complemented full-length information of amino acid sequences revealed the absence of a receiver domain and thereby, confirmed the classification as ERS-type two-component elements. One element is termed HvERS/ETR because partial sequence information generated from contigs 9443, 24802 and missing domains at the C-terminus make a more detailed assignment difficult (Table 2). Two HvETR orthologs that contain the typical receiver domain and build a side branch with ETR proteins from rice in the phylogenetic tree have also been detected in ETCs.

Two transcripts encoding cytokinin receptors bearing the characteristic CHASE domain have been detected in the 454 contigs. Similar to the putative ethylene receptors, deduced amino acid sequences of HvHK4 and HvHK5 reveal a high similarity (77–84%) to the respective rice proteins. Three orthologs of cytokinin-independent HKs were found in the ETC transcriptome. One element, assigned as HvHK1, is grouped together in a side branch with HK2/OHK1 from rice and AHK1 and CKI1 from Arabidopsis, two deduced amino acid sequences cluster together with HK1 from rice and AHK5 from Arabidopsis (Fig. 4A). Translated contig 6273 referred to as barley HvHK3 is identical to ABM92929, which is annotated as functional histidine kinase, but a final assignment remains difficult because parts of the N- and C-terminus compared to the respective rice and Arabidopsis proteins are missing and no receiver domain is predicted.

#### Six genes encoding HPt elements are transcribed in ETCs

Histidine-containing phosphotransfer (HPt) proteins act as intermediates in multi-step phosphorelays by converting signals from HKs to response regulators. Six putative HPt elements have been found in the trancriptome of differentiating ETCs. Among them, three contain a conserved His residue in the HPt domain required for activity in the histidyl-aspartyl phosphorelay whereas three other elements contain Gln and Arg residues instead of His predicting them as pseudo-HPts (Table 2). The monocotyledonous HPt elements seem to be distinct from the dicotyledonous sequences represented by Arabidopsis and build a sidebranch composed of sequences from barley, wheat, rice and maize. Especially the wheat HPt elements show a high similarity to barley isoforms (about 70% for HvHP1 and 2), the closest rice ortholog is OHP2 owing a similarity of around 60% (Fig. 4B). Amino acid sequence of barley HP3 clusters with pseudo-HPts despite the abundance of a conserved His residue in the HPt domain. Two of the three pseudo-HPt elements seem to be more similar to ‘true’ HPt elements in the whole sequence but the His residue is missing; maybe the phylogenetic analysis is blurred by the missing sequence information. In general, the representation of these elements is comparable to those in rice where three pseudo-HPt elements and two real HPt elements have been uncovered in the genome [11].

#### Response regulators transcribed in ETCs can be assigned to three different classes

Response regulators (RRs) can be separated into three subclasses based on sequence comparisons and function: type-A, -B and -C RRs [16]. Additionally, nine and eight pseudo RRs that lack the conserved Asp for phosphorylation and/or contain additional motifs in C-terminal extensions have been detected in the Arabidopsis and rice genome, respectively [17]. Type-A and type-C RRs are composed of a receiver (REC) domain with short N- and C-terminal extensions. Type-B RRs are structurally differing from the other subgroups as they contain long C-terminal extensions with a Myb-like DNA binding domain, assigned as GARP or SANT domain [16].

The three type-A RRs found in the ETC transcriptome show a high similarity to OsRR6, OsRR11 and OsRR9/10 in the amino acid sequence (between 75–94%, Table 2). Interestingly, the maize type-A RRs (ZmTCRR1, 2) whose expression has been detected in basal endosperm transfer cells of maize kernels build a separated cluster in the alignment (Fig. 4C). Intriguing is the high number of type-B RRs in the ETC transcriptome. Eleven sequences encoding type-B elements were present in the 454 contigs. Due to the absence of type-B RR sequence information in barley EST databases and the incomplete genomic backbone most of the RRs of the type-B subgroup could not been complemented to full-length sequences. But as shown in Table 2 a receiver domain and a MYB-like binding domain have been detected in nearly all of the barley amino acid sequences. Apart from nine genes encoding sequences bearing emblematic domains for type-B regulators, two sequences (HvRR4, HvRR12) share the highest homology to RR25 of rice, but fragmental sequence information complicates a final assignment as type-B elements. Remarkably, most of the barley elements transcribed in ETCs build a separated side branch in the type-B family (beside OsRR25), but are part of a monocotyledonous subgroup with OsRR24 and ZmRR9 (Fig. 4D). This might be an indication for the tissue-specifity of these elements and also probable, for the incompleteness of the predicted amino acid sequences possibly resulting in blurred phylogenetic relationships. Three protein sequences (HvPRR3–5) display an additional CCT domain characteristic for Pseudo RRs (PRRs) concomitant to their diverged receiver domains (Table 2). Two other proteins (HvPRR1 and 2) also contain diverged receiver domains. Four isoforms of type-C RRs have been found in the ETC transcriptome with a similarity to the rice counterparts ranging between 53–63%.

### Transcript profiling of TCS elements during ETC differentiation

**Figure 5:**
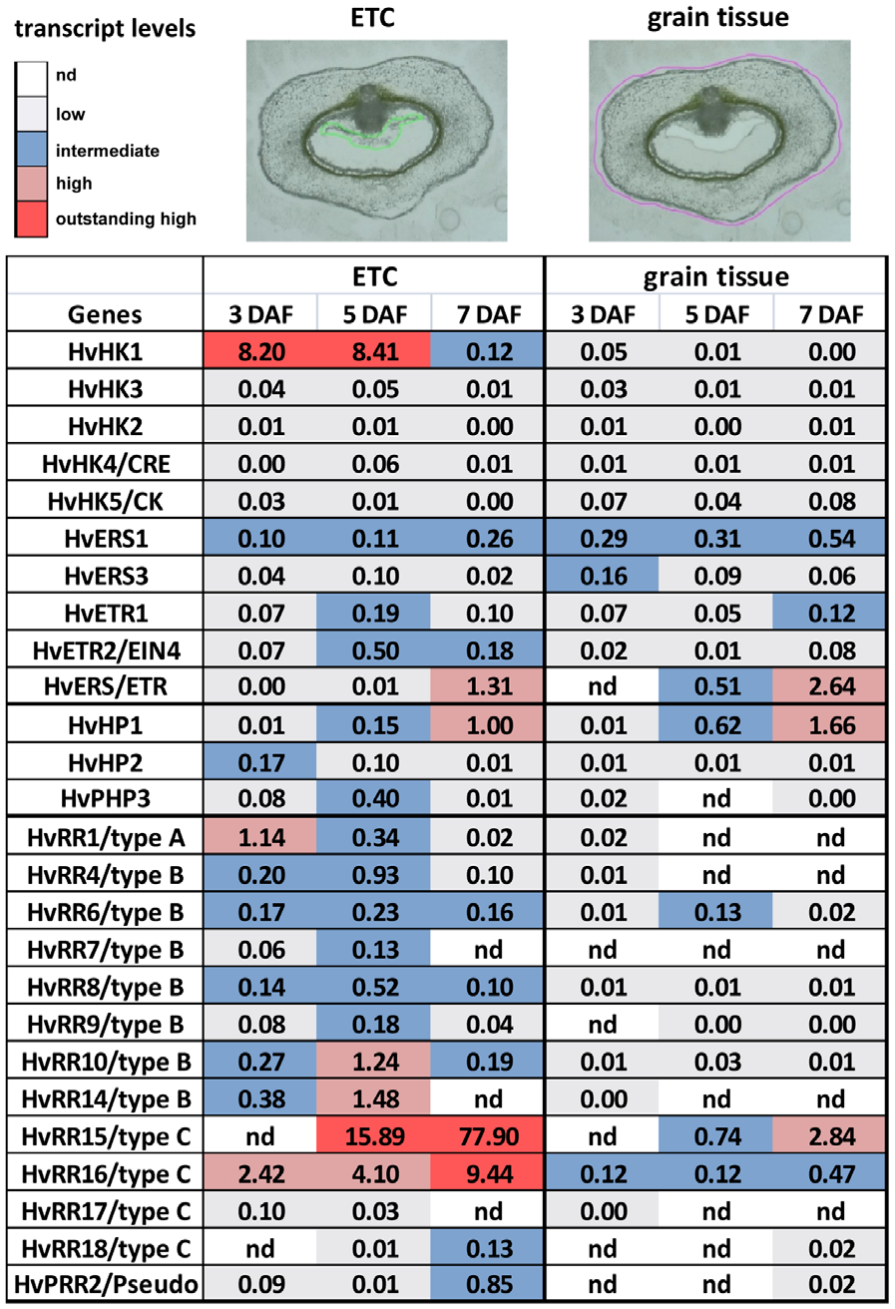
Transcript levels of barley TCS elements in ETCs and grain tissues determined by qRT-PCR analyses. At the top, cross-sections of a barley grain at 3 DAF are given as an example for targets used in qRT-PCR analyses. ETC regions at different stages were isolated by LMPC (encircled in green) and the complete leftovers of the tissue sections, e.g. the whole grain tissues without the ETCs, have been collected separately (encircled in purple). Relative expression levels are illustrated by colour code: white- not detectable (nd), light blue- >0–<0.1/low, blue- 0.1–<1.0/intermediate, pink- 1.0–5.0/high, red- >5.0/very high. Assignment of TCS elements according to Table 2.

To analyze temporal expression profiles of transcripts associated to TCS signal transduction and to get more information about the tissue-specificity we performed qRT-PCR analyses of selected candidates from the gene set presented in Table 2. As targets for the qRT-PCR approach we used aRNA populations of the distinct stages of ETC development which were pooled for 454 sequencing and the complete leftovers of tissue sections at the respective stages (Fig. 5), which will be assigned as grain tissues.

Among the transcripts encoding HKs, *HvHK1* revealed a remarkable high expression at early stages of ETC differentiation (3 DAF and 5 DAF) whereas more or less no expression was detected in the other grain tissues. Transcripts encoding HvETR2 showed also a nearly tissue-specific expression in ETCs but at 5 DAF and with lower expression levels compared to *HvHK1*. One member of the ethylene receptor gene family, which has been assigned as *HvERS/ETR* displayed a high expression in ETCs at 7 DAF, but the expression is not specific for ETCs because of the high expression in other grain tissues. Among the HPt elements, transcriptional activity of HvHP2 peaked during early ETC differentiation (3 DAF) and decreased at later stages (5 and 7 DAF) to the limit of detection whereas almost no expression was observed in other grain tissues. A rather high expression could be detected for *HvPHP3* at 5 DAF. *HvHP1* revealed the strongest expression at 7 DAF the time point when endosperm cellularization has been finished. But the transcriptional activity is not restricted to ETCs (Fig. 5).

The 13 members of the barley response regulator family that have been investigated by transcript profiling revealed distinct expression patterns according to their classification in different subgroups. *HvRR1* belonging to the type-A subgroup showed the highest expression in ETCs at 3 DAF with a decreasing abundance until 5 DAF, thereby also displaying ETC-specific expression. Several genes encoding type-B RRs, namely *HvRR4*, *HvRR7*, *HvRR8*, *HvRR9*, *HvRR10* and *HvRR14*, displayed a maximum of expression in ETCs at 5 DAF with nearly identical profiles. Especially for most candidate genes of the type-B subgroup an expression in the other grain tissues was not detectable or just at the limit of detection. Therefore these genes can be assigned as ETC-specific. Two isoforms of type-C regulators exhibited an extraordinary high expression in ETCs at 7 DAF. *HvRR15* but also *HvRR16* exhibited a strong increase of transcriptional levels from 3 to 7 DAF with expression levels surmounting all other elements of the phosphorelay (Fig. 5).

**Figure 6:**
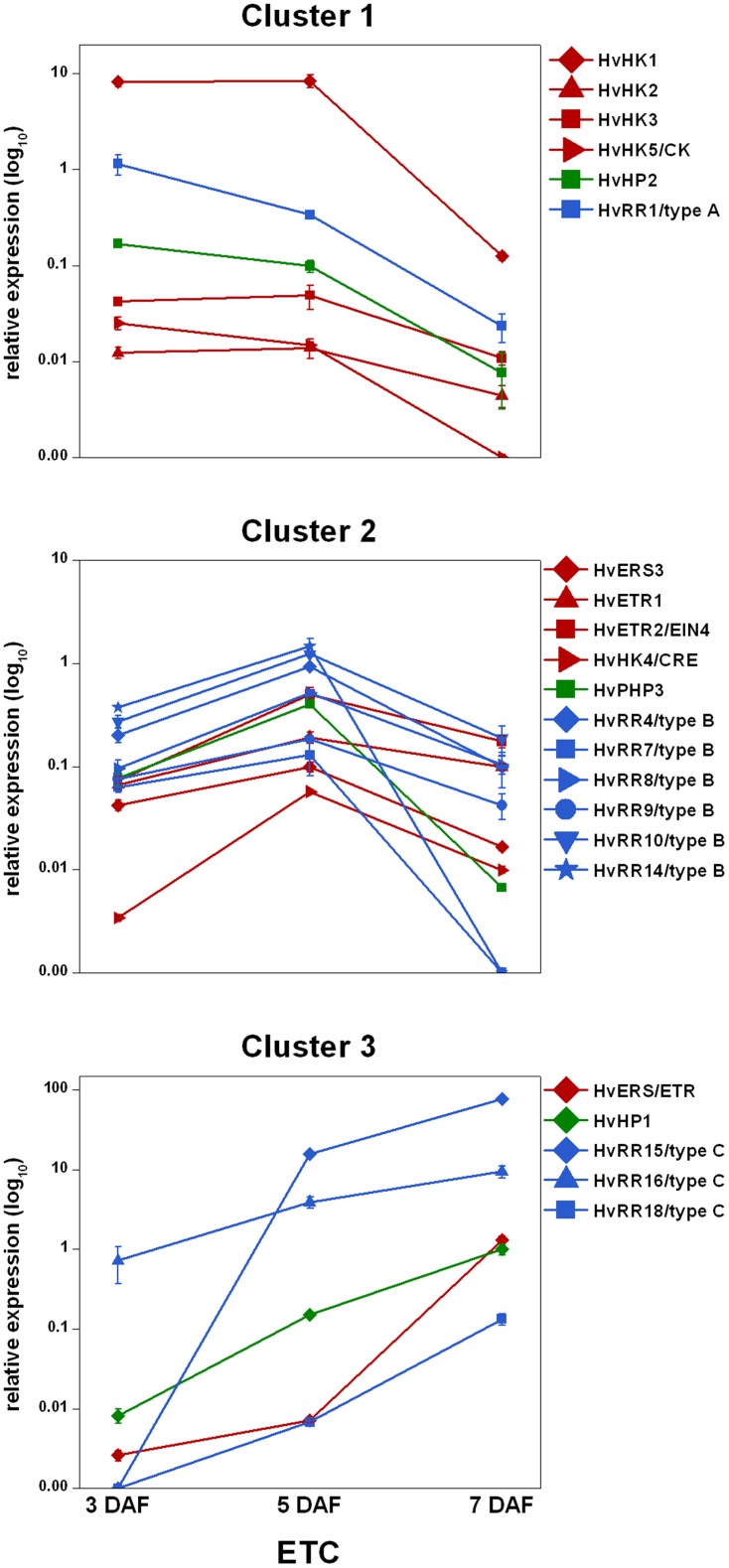
Expression profiles of barley TCS elements in ETCs as determined by qRT-PCR analyses. Expression profiles of candidate genes were grouped by clusters. Cluster 1, genes highly expressed at 3 DAF (and 5 DAF); cluster 2, genes showing a peak of expression at 5 DAF; cluster 3, genes with highest expression levels at 7 DAF. Expression levels are given in the log_10_ scale, values and standard deviations were calculated from three replicates.

For a better illustration of coexpressed members of the different gene families of the TCS, qRT-PCR results were ordered by expression cluster and plotted against a logarithmic scale (Fig. 6). In cluster 1, encompassing genes being expressed before and just at cellularization (3 and 5 DAF) one candidate gene, the cytokinin- and ethylene-independent *HvHK1*, is outstandingly high expressed. Other transcripts encoding histidine kinases (HvHK2, HvHK3, HvHK5) displayed a similar profile, but with rather low expression levels. Coinciding to the transcriptional activity of these HKs, *HvHP2* and *HvRR1* revealed a maximum of expression at that early stage of ETC differentiation. Later on, at 5 DAF numerous type-B RRs showed a characteristic peak in expression (cluster 2). The accumulation of type-B elements is accompanied by a peaking expression of HvERS and HvETR histidine kinases and *HvPHP3* at 5 DAF. Cluster 3 contains genes that show a strong increase of expression from 3 to 7 DAF, the time point when ETCs attain functionality. Beside the outstanding high expression of *HvRR15* and *HvRR16* at 7 DAF, *HvERS/ETR*, *HvHP1* as well as *HvPRR2* appeared to follow this profile.

In summary, it can be noticed that expression profiles of the different elements and components of the TCS indicate specificity for distinct time points in ETC development.

## Discussion

In this report we present results gained from 454 transcriptome sequencing of microdissected ETCs at the syncytial stage, after cellularization and during further ETC maturation. We focussed on LMPC-based isolation of ETCs, because this tissue inside the barley grain is not accessible by other methods and we assumed that this tissue –as the first cellularizing region of the endosperm- has an overall meaning for endosperm differentiation. The approach discovered a high proportion of novel and specialized transcripts. The data suggest a possible crosstalk of hormonal regulation and elements of the two-component signalling (TCS) system as playing an essential role in the differentiation of the ETC region.

### The tissue-specific NGS approach captured new sequence information

One of the most surprising findings of the study was the high amount of tissue-specific transcripts which could not been found in public databases. Alignment to barley cDNA sequence resources revealed that around 40% of the contigs represent new sequence information. Due to the technical challenges, reports about the combination of LMPC and 454 sequencing are missing in plants except one analysis describing the output of a 454 sequencing approach of the maize shoot apical meristem (SAM) [8]. Data generated from maize SAMs revealed a high number of novel sequences, around 30% of the tissue-specific ESTs could not be mapped to the complete EST collection of maize (∼650,000 ESTs). Maize SAMs and barley ETCs have no functional relation. Nevertheless, the methodical approach, including tissue isolation and RNA preparation/amplification, is comparable and gained similar results with regard to new sequence information. During the publication process of the present analysis three studies have been published using a combination of laser microdissection and NGS technologies [18]–[20]. However, the capture of new sequence information was not in the focus of these studies.

Around 8,000 novel ETC-contigs could be functionally annotated or attributed to a putative function by the comprehensive bioinformatics analysis done in this study (Fig. 3). Capturing of new sequence information with supposed relevance for ETC differentiation may help to unravel molecular mechanisms and regulatory networks underlying endosperm differentiation. ETCs are central elements controlling assimilate import into the developing grain and as such, determine agriculturally relevant traits, like grain size and yield. As cereal grains provide the dominant source for the human and animal diet new transcripts putatively implicated in early endosperm differentiation might be of general importance.

### *De novo* transcriptome sequencing suggests a tremendous relevance of the TCS for ETC differentiation

The initial motives to screen the ETC transcriptome for TCS elements came from studies of maize kernels for which a specific expression of two type-A response regulators in maize basal endosperm transfer cells has been shown [9]–[10]. Both RRs are transcriptionally controlled by the MYB-related R1-type transcription factor ZmMRP1 and yeast-two-hybrid studies revealed an interaction of both RRs with ZmHP2. To identify co-expressed genes and further putative interaction partners of the type-A regulators, Muniz et al. profiled other maize TCS elements by qRT-PCR and gained similar expression patterns for some HPt genes. But as the expression data were generated from roughly dissected seed parts, and the upper seed part (without transfer cells) also exhibited high expression levels, a specific localization of these elements in transfer cells remains unclear. Furthermore, expression of TCS genes at very early development, just before and/or after cellularization of transfer cells, was not shown in the frame of this analysis.

Here, combination of NGS technologies with methods allowing the isolation of specific cell types was used to capture the ETC transcriptome at distinct developmental stages. In addition, qRT-PCR analysis of barley TCS elements in microdissected ETCs and the remaining grain tissues revealed distinct temporal transcript profiles in ETCs and allowed us to discriminate tissue-specific expression patterns.

Unexpectedly, 40 genes associated to the TCS are expressed in ETCs, covering all elements and subgroups of this signalling pathway. This is outstanding because it is deduced from genomic information of Arabidopsis and rice that the complete TCS system, including diverged elements, encompasses 54 and 57 genes, respectively [13], [11], implying that nearly the whole barley gene set is expressed in differentiating ETCs. This finding suggests a tremendous relevance of the phosphorelay(s) for ETC initiation and differentiation. The fact that nearly the half of the identified TCS sequences are not present in existing EST databases (Table 2), including HarvEST35 and the full-length cDNA collection of spring barley, supports the specificity of the TCS system for differentiating ETCs.

### A crosstalk of hormonal regulation and TCS elements might be responsible for the differentiation of the ETC region

#### Cellularization of the ETC region is possibly regulated by ABA

During early differentiation of the barley ETC region just at the transition from the syncytial to the cellularized state (3 to 5 DAF), *HvHK1* is highly expressed which is accompanied by a peaking expression of *HvHP2* and *HvRR1* at 3 DAF (Fig. 6, cluster 1). The co-expression of a histidine kinase, a histidine phosphotransfer protein and a type-A response regulator suggests an interaction of these elements in proliferation and initiation of ETC cellularization. A key role in this signal circuit can be assumed for the membrane-bound receptor component, the cytokinin-independent HvHK1. HvHK1 is grouped together in a side branch with AHK1 and CKI1 from Arabidopsis (Fig. 4A). AHK1 was initially identified as a plant osmosensor thereby acting as a positive regulator of salt and drought stress responses [14]. By using loss- and gain-of-function approaches AHK1 has previously been shown to positively affect ABA signalling and to enhance ABA biosynthesis in vegetative and seed tissues under osmotic stress [14]–[15]. Furthermore a regulatory role in seed maturation, including effects on genes required for storage protein accumulation and changed seed moisture content or viability, has been concluded for Arabidopsis. Wohlbach et al. [15] defined an AtMegaCluster to identify genes co-expressed with *AHK1* in different tissues and under different conditions. Several type-A response regulators (*ARR3–4*, *ARR8–9*) were found to depict similar expression profiles probably functioning in the AHK1 phosphorelay. This widely agrees to the expression of type-A *HvRR1* showing a peak of expression at early differentiation whereas at 7 DAF the expression disappeared. Corresponding to the described positive effects of AHK1 on ABA signalling and -biosynthesis this gives first hints to ABA influences on type-A response regulators mediated by a histidine kinase in ETC cellularization.

Based on sequence similarities HvHK1 could also represent an orthologous gene of Arabidopsis CKI1. CKI1 is probably implicated in the regulation of megagametogenesis [21]–[22] correlating to its expression in developing ovules [23] and in development of vascular tissues in Arabidopsis shoots [24]. Genevestigator data specifies expression of *CKI1* exclusively to the chalazal endosperm [25]. Regarding to genevestigator data, *AHK1* is also expressed in the chalazal endosperm but not as specific as *CKI1*. The chalazal endosperm is positioned at the maternal-filial boundary probably facilitating nutrient transfer from the mother plant to the new generation [26]. In analogy to the endosperm coenocyte of cereals the Arabidopsis syncytial endosperm is characterized by rapid proliferation through repeated rounds of nuclear divisions without cytokinesis [26]–[27]. A transcriptome analysis of the syncytial Arabidopsis endosperm using laser capture microdissection identified a plethora of genes preferentially expressed in the endosperm [28]. Interestingly, several genes encoding elements of the TCS system, such as type-B RRs and HPts, have been uncovered in the data set indicating a role for the TCS in the early proliferating Arabidopsis endosperm. Furthermore, a pronounced accumulation of genes implicated in cytokinin (CK) signalling and numerous transcripts involved in ABA and ethylene/ACC signal transduction pathways were deemed to be expressed specifically. Striking is the expression of *NCED6* as a marker gene for ABA biosynthesis implying a role for ABA biosynthesis in early endosperm differentiation of Arabidopsis. This is in line with studies analyzing the influence of ABA and ethylene on endosperm cell division in spikelets of rice [29]. The author concluded a positive correlation of ABA and cell division and subsequently, grain filling. These findings correspond to results obtained from the analysis of the barley endosperm mutant *seg8* and are consistent with the idea that ABA plays an important role in cell cycle regulation during early stages of endosperm development.

In Arabidopsis, most of the type-A ARRs are primary cytokinin response genes as indicated by the rapid transcriptional activation by cytokinin [30]. Genetic analyses showed that several members of the type-A family function as negative regulators of cytokinin signalling by acting in a negative feedback loop to reduce the sensitivity to cytokinin [31]–[32]. The main mechanism regulating the activity of type-A ARRs is proposed to be phosphorylation and protein stability [33]. But it is known that subsets of type-A ARRs are transcriptionally regulated by other transcription factors, such as type-B ARRs for induction [34] or WUSCHEL for repression [35]. Co-occurrence of transcription factor binding sites indicates common mechanisms of transcriptional regulation [36]. Thus, we searched for conserved *cis*-regulatory elements in the promoter regions of *HvHK1*, *HvHP2* and *HvRR1*. From the entity of identified *cis*-elements (Table S5) we focussed on motifs putatively linked to hormone signalling pathways. Despite promoter motifs associated to GA (Agamous, GARE), ethylene (ERE) and SA (ASF1) signalling were also detected, *cis*-elements known to be bound by transcription factors implicated in ABA signalling are highly abundant (Table 3). Analysis of co-occurrence of transcription factor binding sites showed a concerted appearance of motifs (p-values>0.01) recognized by the homeobox gene ATHB5 [37], variations of ABRE motifs (ABRE-like, -related), and the binding site of the specific DREB transcription factor ABI4 of maize [38]. Additional co-occuring *cis*-elements are MYC and MYB binding sites which are bound by the transcriptional activators in ABA signalling AtMYC2 and AtMYB2 [39], binding sites for WRKY71 from rice, a transcriptional repressor of GA signalling [40] and RAV1-A binding sites which are recognized by the VP1/ABI3-related RAV1 protein described to be implicated in ethylene and ABA signalling pathways [41]. Collectively, the results support the hypothesis that ABA influences might be responsible for transcriptional regulation of *HvHK1*, *HvHP2* and *HvRR1* in ETCs.

**Table 3:** Conserved *cis*-regulatory elements putatively implicated in ABA signalling pathways.

		no. of elements			
Motif	Sequence	HvHK1	HvHP2	HvRR1	p-value for co-occurence*
**ATHB**					
ATHB5	CAATTATTG	6	4	1	3.81E-06
**ABRE-like, -related**					
ACGTATERD1	ACGT	2	4	8	3.91E-03
ABRERATCAL	MACGYGB	1	1	3	6.10E-05
ABRELATERD1	ACGTG	—	1	3	
ACGTABOX	TACGTA	—	2	2	
**DRE, DRE-like**					
ABI4-binding site	SYGCYYYY	1	2	3	1.53E-05
LTRECOREATCOR15	CCGAC	1	1	—	
CBF1HV	RYCGAC	—	—	5	
DRE1COREZMRAB17	ACCGAGA	—	—	1	
**DPBF1, 2**					
DPBFCOREDCDC3	ACACNNG	2	—	2	
**MYC**					
MYCCONSENSUSAT	CANNTG	4	6	8	2.44E-04
**MYB**					
MYB1AT	WAACCA	1	2	1	2.44E-04
**WRKY**					
W-box	TTGAC	1	2	—	
WRKY71OS	TGAC	2	6	6	3.91E-03
WBOXHVISO1	TGACT	—	—	1	
**RAV1**					
RAV1-A	CAACA	6	6	4	5.96E-08
RAV1-B	CACCTG	—	1	—	

Regions 1 kb upstream of the predicted start codon of HvHK1, HvHP2 and HvRR1 were screened for known *cis*-elements and interacting transcription factors by using PlantPAN (http://plantpan.mbc.nctu.edu.tw) web-based interface under the default settings (1.0 - core similarity, 0.75 - matrix similarity; detailed results Table S5).

* statistical analysis of co-occurence of transcription factor binding sites in the promoter regions (−1000 bp) of the three genes was conducted by using the PlantPAN tool ‘Gene Group Analysis’ with distance constraint.

**Figure 7:**
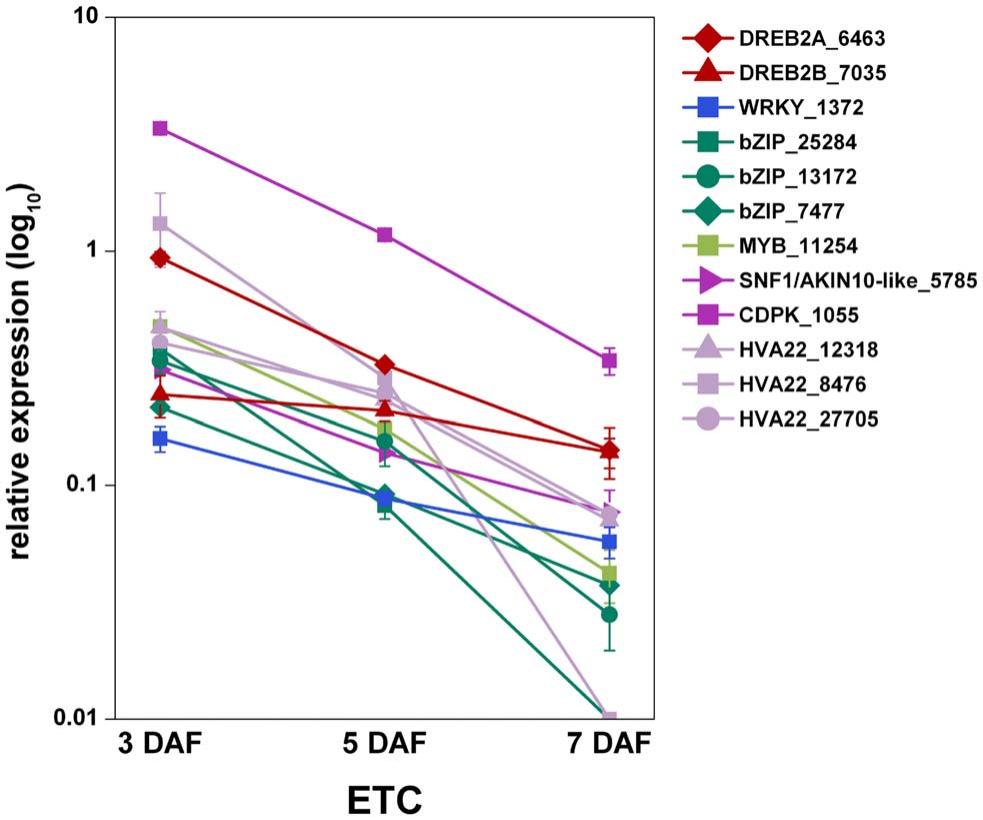
Expression profiles of putative ABA-related transcription factors, -signalling elements and ABA-induced transcripts. Transcript levels in barley ETCs were determined by qRT-PCR analyses. Relative expression is given in the log_10_ scale, values and standard deviations were calculated from three replicates. Transcripts are assigned to gene families according to sequence homology and functional domains, numbers indicate the contig identifier in the 454 transcriptome assembly.

To support the potential role of ABA-related transcription factors in ETC differentiation, the ETC transcriptome was screened for ABA-regulated candidate genes from the transcription factor families depicted in Table 3. Transcript profiling in ETCs by qRT-PCR revealed that three putative bZIP transcription factors, potentially binding the ACTG core motif of ABRE elements, are highly expressed at 3DAF (Fig. 7). Two putative DREB transcription factors (DREB2A, DREB2B), a MYB and a WRKY transcription factor were found to be co-expressed hinting at existing ABA-dependent regulatory networks in ETCs. Furthermore, three members of the HVA22 family, genes which are strongly induced by ABA [42], depict the highest transcriptional activity at 3 DAF with decreasing levels from 5 to 7 DAF. Concomitantly, the expression of candidate genes operating as positive regulators on the ABA signalling pathway, namely a calcium-dependent protein kinase (CDPK) and a SNF1-related protein kinase showing the highest similarity to AKIN10 from Arabidopsis [43], show the same profile (Fig. 7). In summary, expression profiles of ABA signalling elements and ABA-induced transcripts support the idea that ABA might influence ETC cellularization.

Combined with hints for ABA-dependent transcriptional regulation of the HvHK1 phosphorelay, we conclude a crosstalk of ABA and the TCS elements expressed in ETCs during the switch from the syncytial state to cellularization. As the expression of *HvHK1*, *HvHP2* and *HvRR1* clearly decreases and other TCS elements are transcriptionally activated after cellularization, a possible role for the maintenance of the coenocyte and/or the switch to cellularization can be hypothesized for these elements.

#### Differentiation of ETCs might be influenced by ethylene

After cellularization, the ethylene receptors HvETR1, HvETR2 and HvERS3 together with HvPHP3 and six type-B response regulators build a characteristic expression cluster with a peak at 5 DAF (Cluster 2, Fig. 6). Arabidopsis ETR1 and ERS1 have been confirmed to bind ethylene when heterologously expressed in yeast and to modulate ethylene responses in a subtle manner [44]–[45]. A direct but not the major role in ethylene signalling was ascertained for single ethylene receptors [46]. Deduced amino acid sequences of HvETR1, HvETR2 and HvERS3 proteins contain functional histidine kinase domains of ethylene receptors pointing to a general role of ethylene signal transduction during differentiation of ETCs. The expression profiles of type-B RRs follow those of ethylene receptors. As shown in Arabidopsis by different mutational analyses type-B RRs play an essential role in CK signal transduction by acting as positive mediators of CK responses [47]–[49]. Despite these well-known examples, the two putative cytokinin receptors in our data set (HvHK4, HvHK5) show no significant expression in differentiating ETCs (Fig. 6) suggesting that cytokinin regulation might be of low relevance for ETC differentiation. Instead, our data of co-expressed TCS elements in ETCs at 5 DAF allow the assumption that type-B elements interact with HvPHP3 in ETR/ERS-initiated phosphorelays. Additional hints that type-B RRs may contribute to ethylene signalling came from studies of Hass et al. [50] who proposed that the Arabidopsis type-B regulator ARR2 acts downstream of ETR1 and operates independently of confirmed ethylene signalling elements, like CTR1, EIN2 and EIN3. However, these observations are discussed controversely because different results have been obtained by another study analyzing *arr2* mutants [51]. Despite these ambiguous facts yeast two-hybrid analyses with Arabidopsis TCS elements showed potential physical interactions of ETR1 with different HPts, which inturn interact with specific ARRs [52], supporting the view that multi-step phosphorelays could also be involved in signalling pathways of other hormones than cytokinin.

The high number of barley type-B elements displaying overlapping expression profiles in differentiating ETCs can be explained by functional redundancy which has been shown in Arabidopsis revealing that RR type-B phenotypes are only manifested in high-order mutants whereas single mutants do not show visible effects [48]. Intriguing for barley type-B elements is the specificity for ETCs which can be gathered from the widely absent expression in other grain tissues and the fact that nearly all members of this subfamily (except HvRR5, 11) are not present in available EST resources. The possible implication in ethylene-regulated phosphorelays would expand the function of type-B RRs and hints to a crosstalk of different hormone stimuli with TCS elements, thereby reflecting the high complexity of plant signalling networks.

#### ETC maturation could be linked to modified ethylene regulation and type-C response regulators

The transition from ETC differentiation to establishment of active nutrient transfer processes at 7 DAF [53] is characterized by the expression of other types of TCS elements with distinct expression profiles (cluster 3, Fig. 6). Another putative ethylene receptor, HvERS/ETR, shows a strong increase of mRNA abundance at 7 DAF coinciding with the expression of *HvHP1* and two RRs from the C-type (*HvRR15*, *16*). A further main difference is the disappearance of the almost tissue-specific expression as shown for the phosphorelays at earlier stages. The expression is spreading to other grain parts, thereby pointing to a rather general function of these TCS elements in endosperm differentiation. The presence of the sequences in public EST databases (e.g. HarvEST35) supports this view. Of particular interest is the extraordinary high expression of *HvRR15*. As few information about the function of type-C elements is available and no participation in phosphorelays has been shown yet, the possible association or contribution to ethylene signalling pathways represents a further interesting finding. First hints for ethylene effects on ETC differentiation in barley came from transcript profiling of ETCs and cells of the nucellar projection [5] which revealed a pronounced transcriptional activation of enzymes associated to ethylene biosynthesis and catabolism as well as several AP2/EREBP transcription factors in ETCs at 8 DAF. Dibley et al. [54] proposed a role for ethylene signalling in transfer cell induction and development by using an *in vitro* culture system for *Vicia faba* cotyledons. This is in agreement with the finding that the ethylene precursor ACC enhanced the number of cells forming wall ingrowths during TC formation in tomato roots [55]. Microarray-based transcript profiling of barley ETCs at different developmental stages [56] indicated a stimulated expression of SAM synthase and ACC oxidase at 5 DAF and the preferential expression of transcripts encoding ethylene signalling elements, such as several MAP kinases, EIN3 and EIL1 homologues, at 7 DAF. The data suggest ethylene influences on differentiation of barley ETCs but with elements of the ethylene signalling pathway that differ between the beginning of cellularization and continuing maturation. Further analysis of the contribution of type-B and type-C RRs to the described ethylene signal transduction pathways are necessary to shed light on the implication of the two-component signalling system in ETC differentiation and endosperm formation in barley.

In *conclusion* the LMPC-based 454 sequencing approach captured rare and specialized transcripts which have not been accessible in public databases. The general abundance and the specific transcript profiles of TCS elements suggest a pivotal role in distinct steps of ETC development, possibly associated to coordinated hormonal regulation with ABA and ethylene as important components. Transgenic approaches to suppress the expression of peculiar genes belonging to the different phosphorelays are under way and will help to clarify the function of these genes in endosperm differentiation in *planta*. Analysis of protein interactions between TCS elements of specific phosphorelays would be helpful to confirm the conclusions derived from co-expression. Another feature of high interest is the evaluation of the impact of the TCS system on the morphological alterations depicted for the maternal effect barley endosperm mutant *seg8*. We plan to profile the expression of TCS elements identified here in the altered ETC regions of *seg8* to shed light on maternal influences which are possibly reflected in early ETC development.

## Materials and Methods

### Plant material

*Hordeum vulgare* cv. Barke (Saatzucht Josef Breun GmbH & Co. KG, Herzogenaurach, Germany) was grown in greenhouses at 18°C with 16 h light and humidity of 60% air humidity. Flowers are tagged as described in Weschke et al. [53] and caryopses were harvested at 3, 5, and 7 days after flowering (DAF).

### Tissue preparation

Caryopses were frozen in liquid nitrogen and transferred to a cryostat cooled down to −20°C. Using a razor blade, the middle part of the caryopsis was cut out and glued onto the sample plate by using Tissue-Tek® O.C.T™ compound (Sakura Finetek Europe B.V., Zoeterwoude, Netherlands). Sections of 20 µm thickness were cut and immediately mounted on PEN membrane slides (PALM, Bernried, Germany) in the cryostat chamber. PEN membrane slides were stored for 7 days in the cryostat at −20°C until complete dryness. Prior to microdissection, dry cryosections were adapted to room temperature for some minutes. LMPC procedure for isolation of endosperm transfer cells (ETCs) using the PALM® MicroBeam laser system (PALM) has been performed as described [57].

### RNA isolation and mRNA amplification

RNA was extracted from 100 to 200 isolated ETC regions of each developmental stage using the Absolutely RNA Nanoprep Kit (Stratagene) with slight modifications as described [57]. At least 60 ng total RNA was amplified by one round of T7-based mRNA amplification using the MessageAmp aRNA Kit (Ambion) to generate >2 µg of tissue-specific antisense RNA (aRNA) from each developmental stage. Quality assessment of aRNA populations using an Agilent 2100 Bioanalyzer (Agilent Technology) revealed a uniform size distribution from 200 to 2000 nucleotides, with a maximum between 600 and 1000 nucleotides in each of the samples implying few degradation and sufficient RNA quality as the basis for sequencing (Figure S1).

### 454 sequencing

Further processing of the samples and 454 sequencing has been conducted by GATC Biotech (www.gatc-biotech.com). The three aRNA samples were pooled in equal amounts and first strand cDNA synthesis was primed with a N6 randomized primer. After ligation of 454 adapters and PCR-amplification, cDNA was normalized and fractioned cDNA in the size range of 400 to 600 nucleotides was used for GS FLX Titanium sequencing.

### Data processing

Raw data from 454 sequencing runs were vector and quality trimmed by using the SeqClean Software (DFCI Gene Indices Software Tools. http://compbio.dfci.harvard.edu/tgi/software/). Reads shorter than 100 bp were excluded from the analysis. Afterwards the TGICL pipeline was used to generate contigs (consisting of more than one read) and singletons. TGICL pipeline uses megablast [58] for pre-clustering and CAP3 [59] for the assembly process. CAP3 overlap settings were set to 95% identity and 35 bp overlap; all other parameters were set to default.

To evaluate and annotate the generated sequences, BlastX and tBlastX similarity searches against general public databases and species-specific databases with different E-values have been conducted. The databases UniProtKB/Swiss Prot, UniProtKB/TrEMBL (http://www.uniprot.org/) and nr (http://www.ncbi.nlm.nih.gov/) and the plant-specific databases HarvEST assembly 35 (http://www.harvest-web.org/) for *Hordeum vulgare*, TAIR9_cDNA library (http://www.arabidopsis.org/) for *Arabidopsis thaliana*, MIPS_CDS v1.2 library (http://mips.helmholtz-muenchen.de/plant/brachypodium/) for *Brachypodium dystachion* and the Rice Genome Annotation All_cDNA library (http://rice.plantbiology.msu.edu/) for *Oryza sativa* were used for comparison.

For stepwise comparisons and filtering, a perl script (version 5.8.8, http://perl.org) using several BioPerl packages (version 1.6.0, http://bioperl.org) was generated. To identify functional domains in 454 sequences without sufficient similarities to database entries, sequences were translated in all six ORFs and scanned by InterPro database [60].

The RAW data of this study are available at the European Nucleotide Archive (ENA) under the URL: http://www.ebi.ac.uk/ena/data/view/ERP001278.

### Quantitative Real-Time PCR

Unimplemented aRNA used for 454 sequencing and aRNA extracted from the complete leftovers of the tissue sections at each developmental stage were used for qRT-PCR. RNA extraction from 15 to 20 sections of leftovers (grain tissue) and amplification of mRNA was the same as described above.

First strand cDNA was synthesized using SuperScript III (Invitrogen) with random priming according to the manufacturer's instructions. The Power SYBR Green PCR mastermix was used to perform reactions in an ABI 7900 HT Real-Time PCR system (Applied Biosystems, CA, USA). Data were analyzed using SDS 2.2.1 software (Applied Biosystems). Three replications were conducted for each transcript. Expression values were normalized with *HvActin1* (AK365182) and calculated as an arithmetic mean of the replicates. Dissociation curves confirmed the presence of a single amplicon in each PCR reaction. Efficiencies of PCR reactions were determined using LinRegPCR software (http://www.gene-quantification.de/download.html). Values were calculated according to Czechowski et al. [61] and given as relative expression ((1+*E*)^−ΔCt^). A primer list is given in Table S4.

### Identification of *cis*-regulatory elements

Promoter sequences of HvHK1, HvHP2 and HvRR1 were manually identified from genomic sequence information of different barley varieties (http://pgrc.ipk-gatersleben.de/blast/barlex). PlantPAN (http://plantpan.mbc.nctu.edu.tw) web-based interface was used to identify conserved *cis*-elements in the 1-kb upstream regions of the predicted start codon (ATG) under the default settings. The default settings were set to 1.0 for core similarity and 0.75 for matrix similarity. Known transcription factor binding sites were selected from different species (Arabidopsis, barley, maize, rice, tobacco and wheat) and the numbers are given as the sum of elements from both, forward and reverse strand (detailed results in Table S5). Co-occurrence analysis for recognizing combinatorial *cis*-regulatory elements was done by using the ‘Gene group analysis’ function of PlantPAN [36] with the following settings: threshold for co-occurrence support and confidence >90% and distance constraint 100 bp.

### Microscopy

Barley caryopses at different developmental stages were fixated and resin embedded as described [62]. Semi-thin sections (1 µm) were briefly stained with Kristal violet. Bright field and DIC recordings have been made on a Zeiss Axiovert 200 M (Carl Zeiss, Jena, Germany).

## Supporting Information

Figure S1 Quality assessment of RNA isolated from microdissected tissues. Total RNA from LMPC-isolated tissues was extracted and amplified by one round of in vitro transcription (IVT). L, RNA ladder, 1, region of the syncytial endosperm facing the nucellar projection (3 DAF), 2, cellularized ETCs (5 DAF), 3, functional ETCs (7 DAF).
(PPT)

Table S1 Comparison of 454 contigs to general protein and species-specific databases.
(XLSX)

Table S2 Comparison of 454 contigs with HarvEST35 assembly 35.
(XLSX)

Table S3 Multilevel filtering of 454 contigs to public databases.
(XLSX)

Table S4 Primer sequences used for qRT-PCR analyses.
(XLSX)

Table S5 *Cis*-regulatory elements found in the 1-kb upstream regions of genes belonging to expression cluster 1 (Fig. 6).
(XLSX)
