# Supplementary figures and images for: 454 Transcriptome Sequencing Suggests a Role for Two-Component Signalling in Cellularization and Differentiation of Barley Endosperm Transfer Cells
Source: PLoS One. 2012 Jul 25;7(7):e41867. doi: 10.1371/journal.pone.0041867 (PMC3405061; doi:10.1371/journal.pone.0041867)

## Slide 1
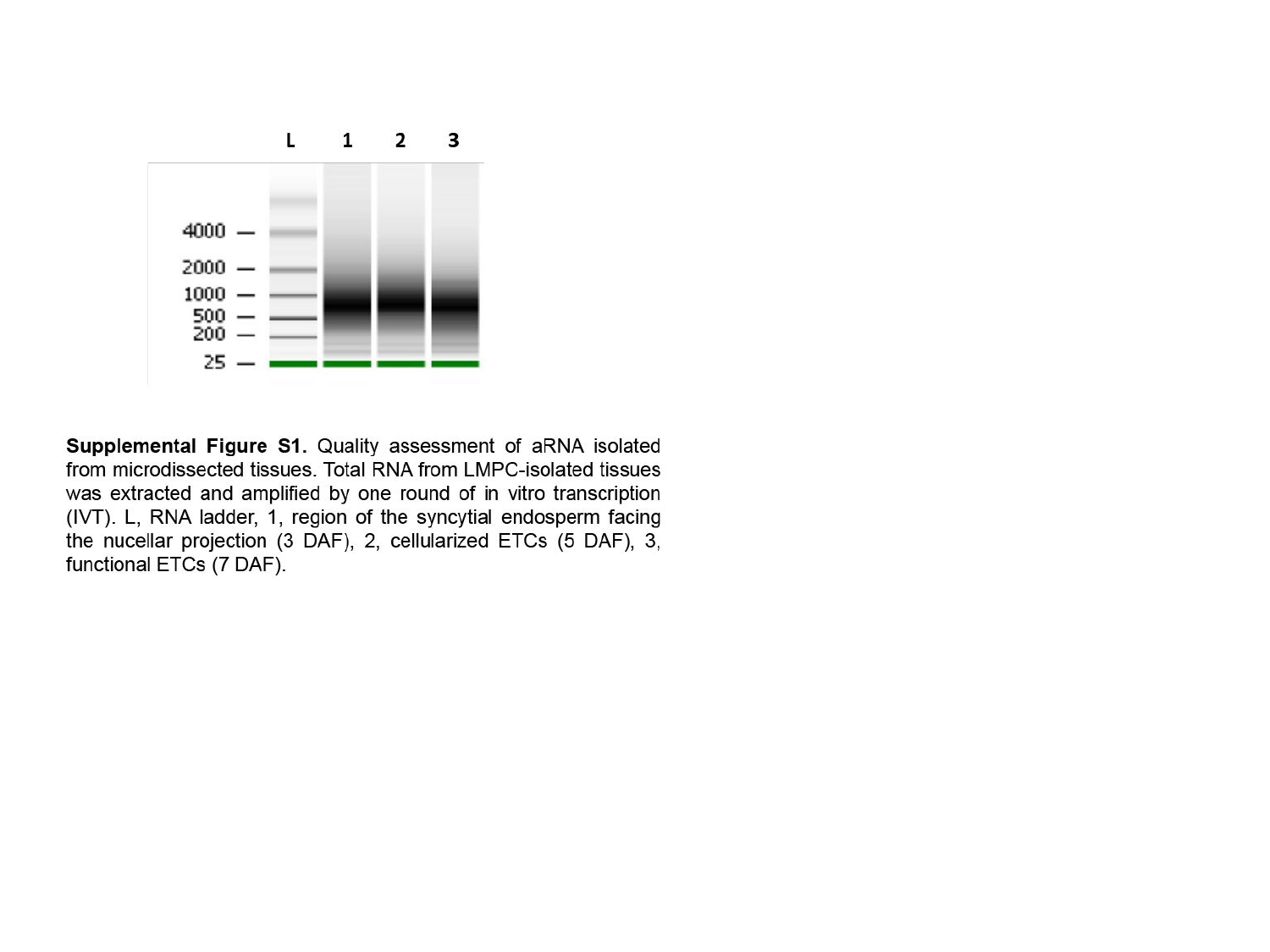

Supplement: Figure S1 — Quality assessment of RNA isolated from microdissected tissues. Total RNA from LMPC-isolated tissues was extracted and amplified by one round of in vitro transcription (IVT). L, RNA ladder, 1, region of the syncytial endosperm facing the nucellar projection (3 DAF), 2, cellularized ETCs (5 DAF), 3, functional ETCs (7 DAF). (PPT) [file pone.0041867.s001.ppt]
